# Differentiation of breast cancer stem cells by knockdown of CD44: promising differentiation therapy

**DOI:** 10.1186/1479-5876-9-209

**Published:** 2011-12-07

**Authors:** Phuc V Pham, Nhan LC Phan, Nhung T Nguyen, Nhung H Truong, Thuy T Duong, Dong V Le, Kiet D Truong, Ngoc K Phan

**Affiliations:** 1 Laboratory of Stem Cell Research and Application, University of Science, Vietnam National University, 227 Nguyen Van Cu, District 5, HCM City, Vietnam; 2 Department of Immunology, Vietnam Military Medical University, 104 Phung Hung, Ha Dong, Ha Noi, Vietnam

**Keywords:** Breast cancer stem cells, Breast cancer cells, CD44, Differentiation, Differentiation therapy, Knockdown

## Abstract

**Background:**

Breast cancer stem cells (BCSCs) are the source of breast tumors. Compared with other cancer cells, cancer stem cells show high resistance to both chemotherapy and radiotherapy. Targeting of BCSCs is thus a potentially promising and effective strategy for breast cancer treatment. Differentiation therapy represents one type of cancer stem-cell-targeting therapy, aimed at attacking the stemness of cancer stem cells, thus reducing their chemo- and radioresistance. In a previous study, we showed that down-regulation of CD44 sensitized BCSCs to the anti-tumor agent doxorubicin. This study aimed to determine if CD44 knockdown caused BCSCs to differentiate into breast cancer non-stem cells (non-BCSCs).

**Methods:**

We isolated a breast cancer cell population (CD44^+^CD24^- ^cells) from primary cultures of malignant breast tumors. These cells were sorted into four sub-populations based on their expression of CD44 and CD24 surface markers. CD44 knockdown in the BCSC population was achieved using small hairpin RNA lentivirus particles. The differentiated status of CD44 knock-down BCSCs was evaluated on the basis of changes in CD44^+^CD24^- ^phenotype, tumorigenesis in NOD/SCID mice, and gene expression in relation to renewal status, metastasis, and cell cycle in comparison with BCSCs and non-BCSCs.

**Results:**

Knockdown of CD44 caused BCSCs to differentiate into non-BCSCs with lower tumorigenic potential, and altered the cell cycle and expression profiles of some stem cell-related genes, making them more similar to those seen in non-BCSCs.

**Conclusions:**

Knockdown of CD44 is an effective strategy for attacking the stemness of BCSCs, resulting in a loss of stemness and an increase in susceptibility to chemotherapy or radiation. The results of this study highlight a potential new strategy for breast cancer treatment through the targeting of BCSCs.

## Background

The existence of breast cancer stem cells (BCSCs) in malignant breast tumors has been demonstrated in many previous studies [1-4]. These stem cells exhibit a range of phenotypes, including CD44^+^CD24^-^, CD44^+^CD24^-/dim^, CD44^+^CD24^-/dim^ESA^+ ^and CD44^+^CD24^-^Lin^- ^[1-4]. These cells possess specific characteristics, such as anti-tumor-drug and radiation resistance [5]. Because they can escape the effects of chemotherapy or radiation therapy, relapse remains a possibility. The resistance of these cells may be mediated by signaling through the Wnt pathway [6]. They also express high levels of anti-apoptotic proteins, such as survivin and Bcl [7], and evidence suggests that alterations in DNA repair and cell cycle kinetics may be involved in their resistance to radiation and chemotherapy [8]. In addition, BCSCs have been shown to be resistant to hormone therapy [9-11]. The discovery of this cancer stem cell population in breast tumors has thus opened up several potential approaches for breast cancer treatment, especially in terms of BCSC-targeting therapy.

The resistance of BCSCs to radiation and chemotherapy means that there is a need to develop agents able to attack this cell population. Because of their stemness, targeting therapies have usually been designed to regulate the self-renewal characteristics, as well as the differentiation of stem cells. Several strategies designed to target BCSCs are currently available, and can be divided into two main groups: those directly targeting BCSCs and those that indirectly targeting BCSCs through the cell microenvironment.

A number of developmental pathways responsible for regulating stemness have been elucidated during the past decade, including Wnt, Notch, and Hedgehog, and several studies have demonstrated that disrupted regulation of these pathways can lead to the development of breast cancer in mice [12-15] and humans [16-18]. HER2 signaling represents one of most significant advances in breast cancer research. Trials of agents targeting HER2, such as trastuzumab and lapatinib, have shown improved overall survival of patients with advanced disease [19] as well as reduced tumor recurrence [20]. Another study found that HER2-targeting agents reduced the BCSC population [19].

However, despite the remarkable clinical efficacy of HER2-targeting agents, a third of HER2-positive tumors do not respond to these agents as well as expected on the basis of their reduced resistance, and almost 50% of patients who respond to HER2-targeted agents relapse within a year [21]. The reason for this phenomenon is unclear. Moreover, nearly 50% of patients are negative for HER2 [22]. Thus the search for new therapeutic strategies continues worldwide.

The adhesion molecule CD44 is a cell surface transmembrane glycoprotein involved in lymphocyte activation, recirculation and homing, adhesion of extracellular matrix, angiogenesis, and cell proliferation, differentiation, and migration [23]. These properties are associated with the pathologic activities of cancer cells. Previous research demonstrated that knockdown of CD44 in BCSCs sensitized them to the anti-tumor drug doxorubicin [24], suggesting that CD44 knockdown affected the stemness or differentiation of these cells.

The current study therefore aimed to investigate the effects of CD44 knockdown on the stemness and differentiation of BCSCs in severe combined immunodeficient (SCID) mice in terms of gene expression, cell cycle, and tumorigenesis, in comparison with breast cancer non-stem cells (non-BCSCs). The results will facilitate the development of BCSC-targeting differentiating gene therapy for breast cancer treatment.

## Materials and methods

### Primary culture of breast cancer cells from malignant breast tumors

Primary culture of breast cancer cells from malignant breast tumors was carried out as previously described [4,24]. Briefly, tumor biopsies were obtained from consenting hospital patients then transferred to our laboratory. Biopsy samples were washed three to four times with phosphate-buffered saline (PBS) supplemented with 1 × antibiotics and an antimycotic (Sigma-Aldrich, St Louis, MO), and homogenized into small fragments (approximately 1-2 mm^3^) using scissors. Homogenized samples were seeded in 35-mm culture dishes (Nunc, Roskilde, Denmark) in M171 medium (Invitrogen, Carlsbad, CA) containing mammary epithelial growth supplement (Invitrogen, Carlsbad, CA), and incubated at 37°C in 5% CO_2_. The medium was refreshed every third day, and the cells were sub-cultured continuously until most cells resembled epithelial-like cells. Ten patients were included in this study, and cancer cells were isolated from all 10 tumors by primary culture.

### Isolation of four cancer cell populations based on CD44 and CD24 expression

All primary cultures were analyzed for the presence of BCSCs by flow cytometry. The samples with the highest percentage of BCSCs were used to isolate four cancer cell populations, based on their expression of CD44 and CD24. CD44^+^CD24^- ^cell populations were classed as BCSCs, while CD44^+^CD24^+^, CD44^-^CD24^+ ^and CD44^-^CD24^- ^cell populations were classed as non-BCSCs or differentiated cells. In subsequent experiments, the term "non-BCSCs" or "differentiated cells" refers to a mixture of the CD44+CD24^+^, CD44^-^CD24^+ ^and CD44^-^CD24^- ^cell populations. These four cancer cell populations were isolated based on their cell surface expression of CD44 and CD24 using a magnetic-activated cell sorting (MACS) system with anti-CD44 and anti-CD24-biotin combined anti-biotin microbeads (Miltenyi Biotec Inc., CA). Positive selection was performed using MS columns, and negative selection using LD columns (Miltenyi Biotec Inc., CA). Cultured cells were detached by trypsin/EDTA 0.25% (Sigma-Aldrich, St Louis, MO). CD44^+^CD24^+ ^cells were isolated in two steps: cells were initially stained with CD44 microbeads and CD44^+ ^cells were collected; the CD44^+ ^cells were then stained with anti-CD24-biotin, followed by anti-biotin microbeads to isolate CD44^+^CD24^+ ^cells. CD44^+^CD24^-^, CD44^-^CD24^+ ^and CD44^-^CD24^- ^cells were similarly isolated by combining positive collections or depletions based on CD44 and CD24 expression, using the same techniques. The phenotypes of all isolated cells were confirmed by flow cytometry using a BD FACSCalibur machine (BD Biosciences, Franklin Lakes, New Jersey) with anti-CD44-phycoerythrin (PE) and anti-CD24-fluorescein isothiocyanate (FITC) monoclonal antibodies (BD Biosciences, Franklin Lakes, New Jersey). Their purities were confirmed by flow cytometry, and samples with > 90% purity were used for further experiments.

### Knockdown of CD44^+^CD24^- ^cells with small hairpin RNA using lentivirus particles

CD44 small hairpin RNA (shRNA) lentivirus particles (Santa Cruz Biotechnology, Inc., Santa Cruz, CA) were stably transfected, according to the manufacturer's instructions. Briefly, CD44^+^CD24^- ^cells were plated on day 1 into 12-well plates with complete medium (DMEM/F12 supplemented with 10% fetal bovine serum and 1 × antibiotic-mycotic) and incubated overnight. The medium was replaced on day 2 with fresh complete medium supplemented with 5 μg/ml polybrene (Sigma-Aldrich, St Louis, MO) for 6 h, after which 20 μl MEM with 25 mM HEPES containing 10^5 ^infectious units of virus were directly added into the culture. The plate was shaken to mix the virus particles and incubated overnight at 37°C in 5% CO_2_. The medium was changed on day 3 to fresh complete medium without polybrene. Successfully transduced cells were selected by culturing in complete medium supplemented with 10 μg/ml puromycin dihydrochloride (Sigma-Aldrich, St Louis, MO) for 12 h, followed by 5 μg/ml puromycin dihydrochloride for 1 week. CD44 knockdown BCSCs were confirmed by determination of CD44 expression by flow cytometry and immunocytochemistry. Samples with > 90% purity were used for further experiments for evaluating tumorigenesis in SCID mice and investigating gene expression and cell cycle.

### Flow cytometry

Cells were washed twice in PBS supplemented with 1% bovine serum albumin (Sigma-Aldrich, St Louis, MO). The cell surface Fc receptor was blocked using IgG (Santa Cruz Biotechnology, Inc., Santa Cruz, CA) on ice for 15 min. Cells were stained for 30 min at 4°C with anti-CD44-PE and anti-CD24 FITC monoclonal antibodies (BD Biosciences, Franklin Lakes, New Jersey). After washing, cells were analyzed using a FACSCalibur flow cytometer (BD Biosciences, Franklin Lakes, New Jersey) using CellQuest Pro software at 10,000 events.

### Gene expression analysis

Ten random colonies formed after plating CD44 knockdown BCSCs at low density were used for the analysis of gene expression. To evaluate the differentiated status of BCSCs, expression of 15 genes related to the properties of cancer stem cells and cancer/normal cells, as well as some genes related to signaling pathways over-expressed in cancer stem cells, were analyzed in comparison with BCSCs and non-BCSCs. Glyceraldehyde 3-phosphate dehydrogenase (GAPDH; [Genebank:NM_002046]) was used as an internal control for all experiments. All primers used in this study were designed using Primer Blast software (NCBI). Primer pairs were chosen to give polymerase chain reaction (PCR) products of 100-350 bp. The universal primer for each forward and reverse primer was then added. The universal primer sequence was suggested by the manufacturer, using the GenomeLab GeXP genetic analysis system (Beckman-Coulter, Brea, California). All primer sequences are listed in Table 1. All primers were checked for specificity and working status by *in silico *PCR and *in vitro *reverse-transcription PCR using a universal RNA template (Clontech, CA, USA). Only primer pairs that gave the intended PCR products were used in subsequent experiments. Two multiplex PCR reactions were used to evaluate the change in stemness: one multiplex with 17 genes included *Bcl-2 *[Genebank:NM_000633], *Fos *[Genebank: NM_005252], *ICAM1 *[Genebank:NM_000201], *CCND1 *[Genebank: NM_053056], *MMP7 *[Genebank:NM_002423], *Myc *[Genebank:NM_002467], *PRKCE *[Genebank:NM_005400], *TP53 *[Genebank:NM_000546], *VCAM1 *[Genebank:NM_001078], *IL4R *[Genebank:NM_000418], *PTCH1 *[Genebank:NM_000264], *HSPB1 *[Genebank: NM_001540], *PTGS2 *[Genebank: NM_000963], *HSF1 *[Genebank:NM_005526], *LEF1 *[Genebank:NM_016269], *TCF7 *[Genebank:NM_003202], and *FASN *[Genebank:NM_004104] and the other with five genes included *Muc-1 *[Genebank:NM_002456], *cyclin E2 *[Genebank:NM_004702], *EGFR *[Genebank:NM_005228], *Myc *[Genebank:NM_002467], and *cyclin D1 *[Genebank:BC001501].

**Table 1:** Primer sequences used in this research.

Gene	Sequence	GeXP PCR product (bp)
**Multiplex 1: 17 genes (+ GAPDH and KanR)**		

Bcl-2 (B-cell lymphoma 2)	**F:**AGGTGACACTATAGAATATGGAACTGTACGGCCCCAGCA	140
		
[Genebank:NM_000633]	**R:**GTACGACTCACTATAGGGAAGGGTGATGCAAGCTCCCACCA	

Fos (FBJ murine osteosarcoma viral oncogene homolog)	**F:**AGGTGACACTATAGAATAGTTATAAAAGCAGTGGCTGCGGC	153
		
[Genebank: NM_005252]	**R:**GTACGACTCACTATAGGGAAGCACGGTCACTGCTCGTTCG	

ICAM1 (intercellular adhesion molecule 1)	**F:**AGGTGACACTATAGAATAGTGCTATTCAAACTGCCCTGA	169
		
[Genebank:NM_000201]	**R:**GTACGACTCACTATAGGGAGCGTAGGGTAAGGTTCTTGC	

CCND1 (cyclin D1)	**F:**AGGTGACACTATAGAATAGCATGTTCGTGGCCTCTAAG	177
		
[Genebank: NM_053056]	**R:**GTACGACTCACTATAGGGACAGGTTCCACTTGAGCTTGTT	

MMP7 (matrix metalloproteinase-7)	**F:**AGGTGACACTATAGAATATTGGCTTTGCGCGAGGAGCT	181
		
[Genebank:NM_002423]	**R**:GTACGACTCACTATAGGGACTGCTACCATCCGTCCAGCGT	

Myc (V-myc myelocytomatosis viral oncogene homolog (avian))	**F:**AGGTGACACTATAGAATACTCTCCTTGCAGCTGCTTAGA	187
		
[Genebank:NM_002467]	**R:**GTACGACTCACTATAGGGACCTCGTCGCAGTAGAAATACG	

PRKCE (Protein kinase C epsilon type)	**F:**AGGTGACACTATAGAATAAAGGTCCCTACCTTCTGCGA	197
		
[Genebank:NM_005400]	**R:**GTACGACTCACTATAGGGACCAGTACTTTGGCGATTCCT	

TP53 (Cellular Tumor Antigen p53)	**F:**AGGTGACACTATAGAATATTCCCTGGATTGGCAGCCAGACTG	201.5
		
[Genebank:NM_000546]	**R:**GTACGACTCACTATAGGGATCCATTGCTTGGGACGGCAAGG	

VCAM1 (vascular cell adhesion molecule 1)	**F:**AGGTGACACTATAGAATAAGGTGACGAATGAGGGGACCACATC	215.4
		
[Genebank:NM_001078]	**R:**GTACGACTCACTATAGGGAAGCCTCCAGAGGGCCACTCAAA	

IL4R (IL-4 receptor)	**F:**AGGTGACACTATAGAATAAAGTGGCACAACTCCTACAGG	231
		
[Genebank:NM_000418]	**R:**GTACGACTCACTATAGGGACCCTGAGCATCCTGGATTATT	

PTCH1 (Protein patched homolog 1)	**F:**AGGTGACACTATAGAATAGCCTTCGCTCTGGAGCAGATTT	240
		
[Genebank:NM_000264]	**R:**GTACGACTCACTATAGGGAGTTGGTCTCGAGGTTCGCTGCTT	

HSPB1(heat shock 27 kDa protein 1)	**F:**AGGTGACACTATAGAATAGCACACTGACCGTGGAGGCC	241
		
[Genebank: NM_001540]	**R:**GTACGACTCACTATAGGGAGAACACACAGGTGGCGGGGG	

PTGS2 (prostaglandin-endoperoxide synthase 2)	**F:**AGGTGACACTATAGAATACAGCTCCACAGCCAGACGCC	249
		
[Genebank: NM_000963]	**R:**GTACGACTCACTATAGGGATCCTGTCCGGGTACAATCGCACT	

HSF1 (Heat shock factor protein 1)	**F:**AGGTGACACTATAGAATAGCCTTCCTGACCAAGCTGTGGACC	271
		
[Genebank:NM_005526]	**R:**GTACGACTCACTATAGGGATCTCTCTGGCTTGACCAGGCCG	

GAPDH (Glyceraldehyde 3-phosphate dehydrogenase)	**F:**AGGTGACACTATAGAATAAAGGTGAAGGTCGGAGTCAA	277.2
		
[Genebank:NM_002046]	**R:**GTACGACTCACTATAGGGAGATCTCGCTCCTGGAAGATG	

LEF1 (Lymphoid enhancer-binding factor-1)	**F:**AGGTGACACTATAGAATAGGTGCAGCCATCCCATGCGGT	290.2
		
[Genebank:NM_016269]	**R:**GTACGACTCACTATAGGGAAGGGTTGCCTGAATCCACCCGTG	

TCF7 (Transcription factor 7)	**F:**AGGTGACACTATAGAATAGGCGAGGAGCAGGACGACAAGAG	305
		
[Genebank:NM_003202]	**R:**GTACGACTCACTATAGGGATTTGTACATGCCGCTGGTGCAC	

FASN (Fatty acid synthase)	**F:**AGGTGACACTATAGAATAGAGTCGGAGAACTTGCAGGAGT	307
		
[Genebank:NM_004104]	**R:**GTACGACTCACTATAGGGAGTGTGTTCCTCGGAGTGAATCT	

KanR (Kanamycin resistance)	**F:**AGGTGACACTATAGAATAATCATCAGCATTGCATTCGATTCCTGTTTG	325.4
		
	**R:**GTACGACTCACTATAGGGAATTCCGACTCGTCCAACATC	

**Multiplex 1: 5 genes (+ GAPDH and KanR)**		

Muc-1 (Mucin 1, transmembrane, transcript variant 1)	**F:**AGGTGACACTATAGAATAGACGTCAGCGTGAGTGATGT	177.7
		
[Genebank:NM_002456]	**R:**GTACGACTCACTATAGGGAGACAGCCAAGGCAATGAGAT	

Cyclin E2 (Cyclin E2 (CCNE2), transcript variant 3)	**F:**AGGTGACACTATAGAATAGAGCCCGAAGAGCACTGAAAAACC	194.6
		
[Genebank:NM_004702]	**R:**GTACGACTCACTATAGGGAGAGGAATTGGCTAGGGCAATCAA	

EGFR (EGF receptor, transcript variant 1)	**F:**AGGTGACACTATAGAATAGAAAGGCAGCCACCAAATTA	208.4
		
[Genebank:NM_005228]	**R:**GTACGACTCACTATAGGGAACTATCCTCCGTGGTCATGC	

Myc (V-myc myelocytomatosis viral oncogene homolog (avian))	**F:**AGGTGACACTATAGAATAGAGCAACGTCTCCACACATCAGCAC	217
		
[Genebank:NM_002467]	**R:**GTACGACTCACTATAGGGAGAGTTTTGTGTGTTCGCCTCTTGAC	

Cyclin D1 (G1/S-specific cyclin-D1)	**F:**AGGTGACACTATAGAATACGTGGCCTCTAAGATGAAGG	252.5
		
[Genebank:BC001501]	**R:**GTACGACTCACTATAGGGATGCGGATGATCTGTTTGTTC	

GAPDH (Glyceraldehyde 3-phosphate dehydrogenase)	**F:**AGGTGACACTATAGAATAAAGGTGAAGGTCGGAGTCAA	277.2
		
[Genebank:NM_002046]	**R:**GTACGACTCACTATAGGGAGATCTCGCTCCTGGAAGATG	

KanR (Kanamycin resistance)	**F:**AGGTGACACTATAGAATAATCATCAGCATTGCATTCGATTCCTGTTTG	325.4
		
	**R:**GTACGACTCACTATAGGGAATTCCGACTCGTCCAACATC	

F: Forward; R: Reverse.

RNA was isolated from all cell samples using an RNA isolation kit (Fermentas, Glen Burnie, Maryland, USA). Gene expression levels of 15 genes involved in drug resistance, cell cycle and signaling pathways were assayed using the capillary GenomeLab GeXP genetic analysis system (Beckman-Coulter, Brea, California). A multiplex panel was designed to assess the genes. In addition to the genes of interest, each panel contained an internal control gene (kanamycin resistance, KanR) and a normalization gene (GAPDH). cDNA was synthesized from 500 ng total RNA using the GenomeLab GeXP Start Kit (Beckman-Coulter, Brea, California). PCR and multiplex detection were performed according to the manufacturer's instructions. GeXP data were analyzed after normalization of all genes of interest against the geometric mean of the normalization gene.

### Cell cycle assay

Cell cycle analysis was carried out according to the following protocols. Cells from each group were washed twice in PBS and fixed in cold 70% ethanol for at least 3 h at 4°C. Cells were then washed twice in PBS and stained with 1 ml of PI (20 μg/ml). A 50-μl volume of RNase A (10 μg/ml) was added to the samples and incubated for 3 h at 4°C. Stained cells were analyzed by flow cytometry using CellQuest Pro software (BD Biosciences, Franklin Lakes, New Jersey).

### Tumorigenesis assay

Non-obese diabetic (NOD)/SCID mice (5-6 weeks old) (NOD.CB17-Prkdcscid/J) (Charles River Laboratories) were used in this research. Four experiments were performed to evaluate the effects of CD44 knockdown on the tumorigenic potential of BCSCs. In the first experiment, BCSCs were injected into 12 mice with three mice/dose at 10^6^, 10^5^, 10^4 ^and 10^3 ^cells/mouse. Similarly, CD44 knockdown BCSCs and non-BCSCs were injected at the same doses in the second and third experiments. The fourth experiment comprised negative control mice injected with PBS. The first and second, and third and fourth experiments, respectively, were performed using the right and left sides of the same mice. All mice were housed in clean cages and maintained according to institutional guidelines on animal welfare. Mice were followed up for 2 months to detect tumors.

### Statistical analysis

All experiments were performed in triplicate. Differences between mean values were assessed by *t*-tests and analysis of variance. A *P *value of ≤ 0.05 was considered to be significant. Data were analyzed using Statgraphics software (v 7.0; Statgraphics Graphics System, Warrenton, VA).

## Results

### Isolation and culture of BCSCs and non-BCSCs

**Figure 1:**
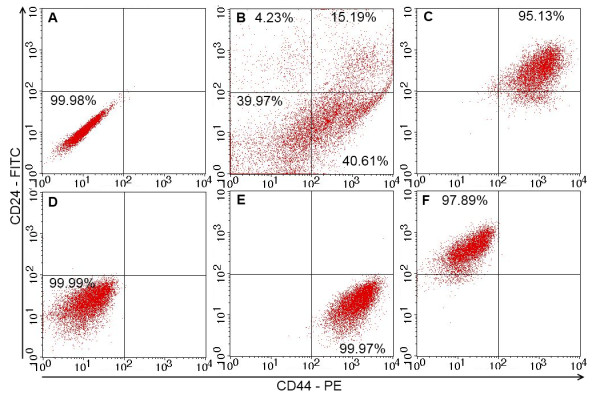
**Expression of CD44 and CD24 in four different cell populations analyzed by flow cytometry**. (A) Unstained cells. The breast cancer cell population (B) was sorted into four populations: CD44^+^CD24^+ ^(C), CD44^-^CD24^+ ^(D) and CD44^-^CD24^- ^(E), CD44^+^CD24^- ^(BCSCs) (F). All sorted cell populations exhibited high degrees of purity.

**Figure 2:**
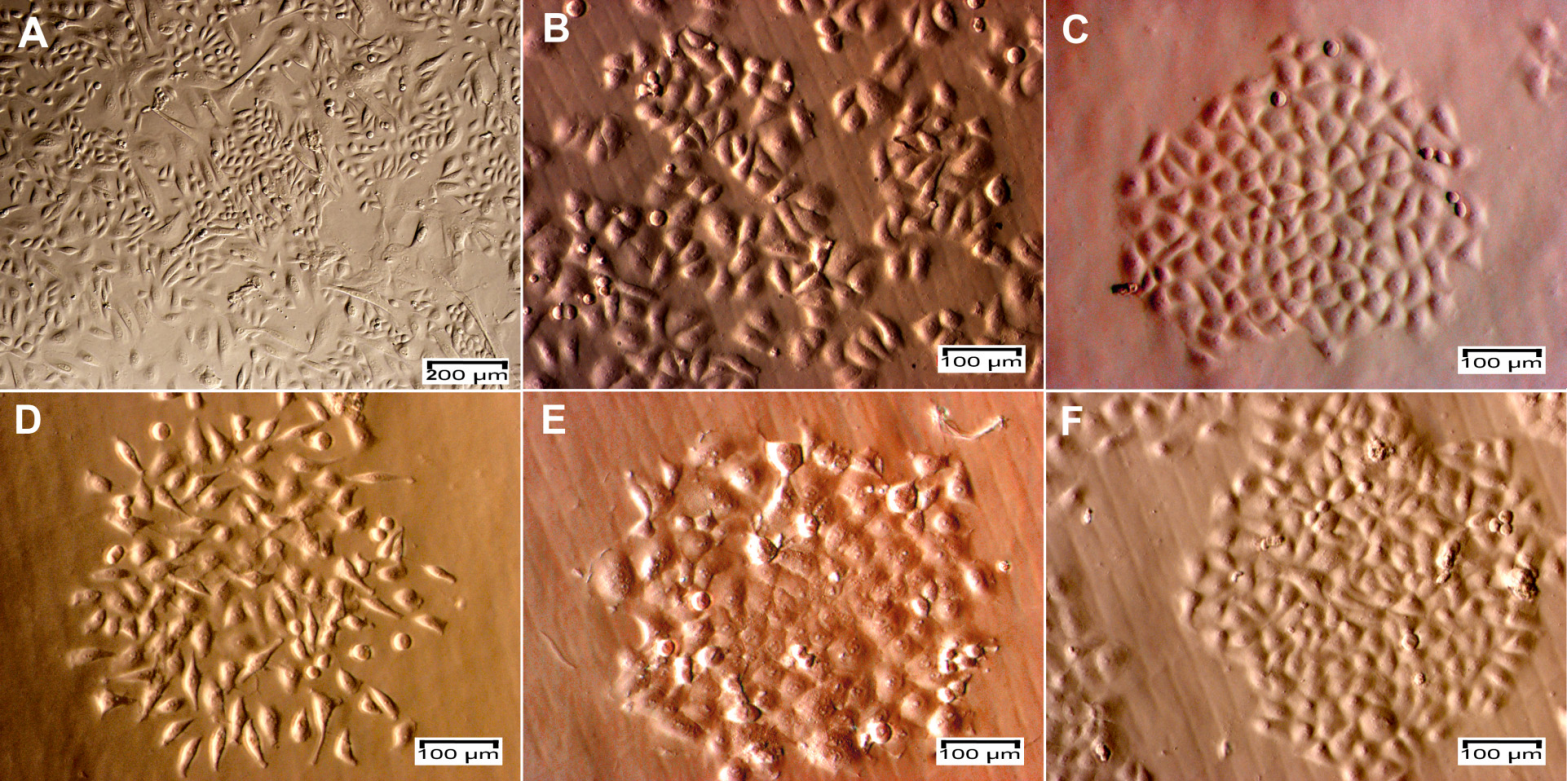
**Primary cells, breast cancer cells and four sub-populations of CD44^+^CD24^+^, CD44^+^CD24^-^, CD44^-^CD24^- ^and CD44^-^CD24^+ ^cells**. Cells expanding from the tissue (A) showed at least two different shapes (epithelial and stromal) and became homogeneous after sub-culturing (B). These cells were sorted into four sub-populations of CD44^-^CD24^+ ^(C), CD44^-^CD24^- ^(D), CD44^+^CD24^- ^(E) and CD44^+^CD24^+ ^cells (F).

Primary cultures were derived from 10 breast tumor samples, including eight well-developed tumors with many cells expanding from the tissues and two samples that were infected with micro-organisms. The primary cultures were sub-cultured to produce a large number of cells, which were then evaluated for the presence of BCSCs. BCSCs comprised 4.32 ± 1.78% of all the samples (range 2.51-7.09%) (Figure 1A, B). The sample with the highest BCSC population was used to isolate four populations based on the expression of CD44 and CD24 markers (CD44^+^CD24^-^, CD44^+^CD24^+^, CD44^-^CD24^+^, and CD44^-^CD24^- ^cells) by magnetic cell sorting. The purities of the isolated cell populations assessed by flow cytometry ranged from 95.14-99.99% (Figure 1C-F). The four cultured cell populations had homogeneous shapes (Figure 2) and were cultured and subcultured to produce sufficient cells for further experiments.

### Expression of CD44 in CD44 knockdown BCSCs

**Figure 3:**
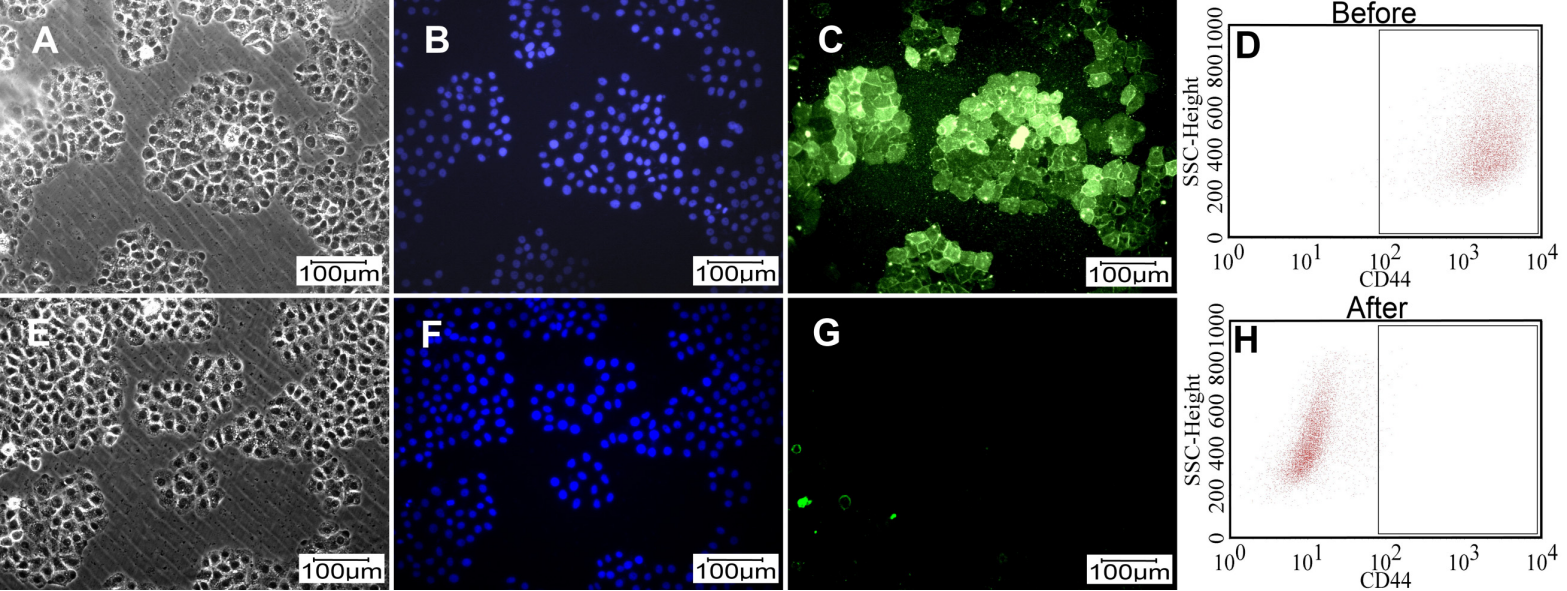
**CD44 expression before (A, B, C and D) and after CD44 knockdown by shRNA (E, F, G and H) combined with selection using puromycin dihydrochloride for 1 week**. Cells were evaluated by immunocytochemistry after staining with anti-CD44 and fluorescein-isothiocyanate (FITC)-conjugated secondary antibody and were observed under a monochromatic fluorescence microscope (Carl Zeiss, Oberkochen, Germany) with white light, Hoechst 33342 and a FITC filter (A and E, B and F, C and G, respectively) and by flow cytometry after staining with anti-CD44-FITC (D and H).

Following shRNA lentivirus transfection and selection with puromycin for 1 week, CD44 knockdown BCSCs showed decreased CD44 expression compared with BCSCs before knockdown, as demonstrated by immunocytochemistry and flow cytometry (Figure 3). Thus CD44 shRNA lentivirus combined with puromycin selection efficiently silenced CD44 mRNA expression in treated cells. Protein quantification by flow cytometry demonstrated that the percentage of CD44-positive cells in BCSCs before and after CD44 knockdown was reduced from 96.32% ± 3.33% to 0.12% ± 0.03% (n = 3) (Figure 3D, H). This level of suppression was greater than that achieved in a previous study using transfection of small interfering RNA (siRNA) [24].

### Gene expression in CD44 knockdown BCSCs compared with BCSCs and non-BCSCs

**Figure 4:**
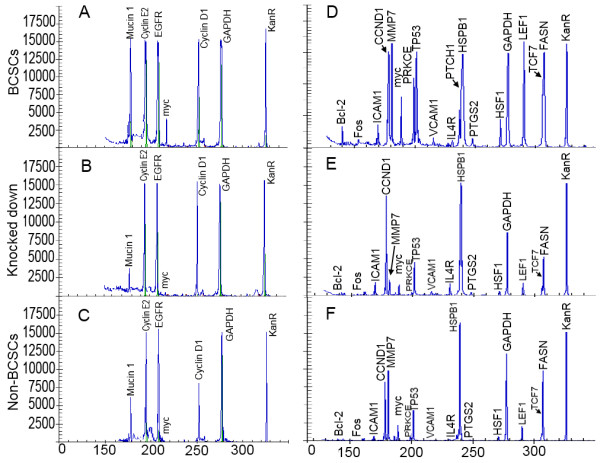
**Similar gene expression patterns in CD44 knockdown BCSCs (B, E) and non-BCSCs (C, F) compared with BCSCs (A, D), in genes related to cell viability, proliferation, metastasis and anti-tumor drug resistance, analyzed using a genetic analysis system (GeXP, Beckman-Coulter, Brea, California)**. Knocked down: CD44 knockdown BCSCs.

**Figure 5:**
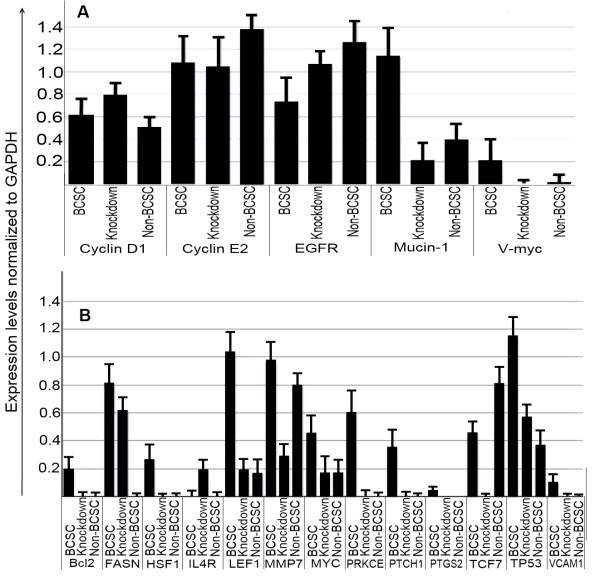
**Expression levels of genes related to metastasis (A), cell viability, proliferation and anti-tumor drug resistance (B) in BCSCs, non-BCSCs and CD44 knockdown BCSCs normalized to GAPDH**. Knockdown: CD44 knockdown BCSCs.

The expression of important genes related to stemness, anti-tumor drug resistance, and metastasis in BCSCs was altered in CD44 knockdown BCSCs, as shown in Figures 4 and 5. *Muc-1*, *MMP9*, and *Myc *expression levels were strongly reduced by CD44 knockdown, bringing them in line with levels in non-BCSCs. Levels of several other genes such as *EGFR *and *cyclin D1 *also fell. High Bcl-2 expression in BCSCs is particularly associated with chemoresistance, and its expression also decreased to the level in non-BCSCs after knockdown of CD44. The expression of genes related to stemness, such as *LEF1*, also decreased. *LEF1*, *TCF7*, and *Myc *are members of the Wnt signaling pathway; *Bcl-2*, *MMP7*, and *Myc *are members of the PI3K/AKT signaling pathway; *HSF1*, *TP53*, and *MYC *are members of the Stress pathway; and *PTCH1*, *PKRCE*, *PTGS2*, and *IL4R *are members of the Hedgehog signaling pathway. Expression of all these genes was reduced to levels similar to those seen in non-BCSCs.

### Cell cycle in CD44 knockdown BCSCs compared with BCSCs and non-BCSCs

**Figure 6:**
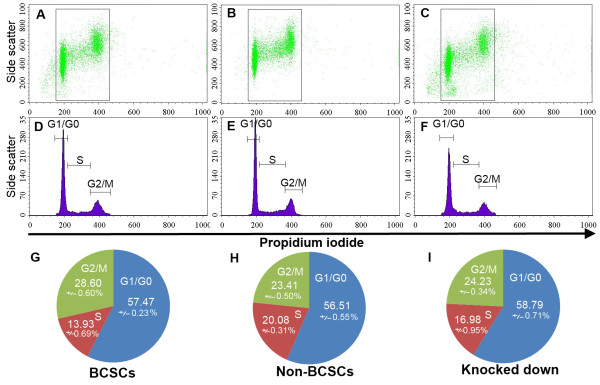
**Similar cell cycles in non-BCSCs (B, E, H), and CD44 knockdown BCSCs (C, F, I) cells compared with BCSCs (A, D, G)**. G1/G0 phase was relatively unchanged, but G2/M phase decreased and S phase increased in CD44 knockdown BCSCs.

The cell cycle was affected by knock-down of CD44, as shown in Figure 6. The percentage of cells in G2/M phase was significantly higher in BCSCs compared with non-BCSCs (28.60 ± 0.60% vs. 23.41% ± 0.50%, *P *< 0.05) while the number of cells in S phase was lower (13.93 ± 0.69% vs 20.08 ± 0.31%, *P *< 0.05). In contrast, the numbers of cells in G1/G0 phase were similar in BCSCs and non-BCSCs (57.47 ± 0.23% vs 56.51 ± 0.55%, *P *> 0.05). G2/M phase and S phase in CD44 knockdown BCSCs approached those in non-BCSCs. G2/M phase in CD44 knockdown BCSCs decreased and was similar to non-BCSCs (24.23 ± 0.34% vs 23.41 ± 0.50%, respectively) while S phase increased from 13.93 ± 0.69% in BCSCs to 16.98 ± 0.95% in CD44 knockdown BCSCs, compared with 20.08 ± 0.31% in non-BCSCs (Figure 6). The percentages of cells in G1/G0 phase in BCSCs, non-BCSCs and CD44 knockdown BCSCs were similar. These results suggest that CD44 knockdown decreased proliferating ability and extended S phase to increase the similarities with non-BCSCs.

### Tumorigenesis of CD44 knockdown BCSCs compared with BCSCs and non-BCSCs in NOD/SCID mice

**Figure 7:**
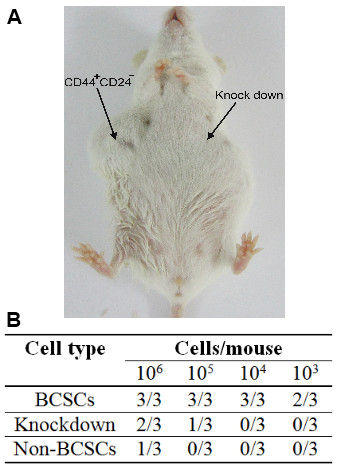
**Tumorigenic capacities of CD44 knockdown BCSCs, BCSCs and non-BCSCs in NOD/SCID mice**. CD44 knockdown BCSCs and non-BCSCs were similar, but there was a significant difference in tumorigenic capacity between CD44 knockdown BCSCs and BCSCs (B). Injection of 10^6 ^BCSCs caused large tumors (right), while 10^6 ^CD44 knockdown BCSCs failed to produce any tumors (left) (A).

The tumor-causing potential of the cells was evaluated to assess the differentiated phenotype after CD44 knockdown. BCSCs caused tumors in 66.67% (2/3) of mice with 10^3 ^cells, while 10^6 ^non-BCSCs were needed to cause tumors in 25% of mice (1/3). The tumor-causing potential was reduced in the CD44 knockdown BCSCs, with doses of 10^4 ^cells causing tumors in 0% of mice, compared with 100% of mice before CD44 down-regulation (Figure 7). At doses of 10^5 ^grafted cells, BCSCs were capable of generating tumors in up to 100% of mice (3/3), compared with only 33.33% (1/3) of mice in the case of CD44 knockdown BCSCs. Figure 7A shows that injection with 10^6 ^BCSCs caused large tumors (on the right), while 10^6 ^CD44 knockdown BCSCs failed to produce any tumors (on the left). This suggests that knockdown of CD44 caused differentiation and loss of the stemness characteristics of BCSCs.

## Discussion

The effectiveness of breast cancer treatment currently remains low. This may be attributable to the existence of a small population of cancer stem cells with high resistance to chemotherapy and radiation therapy, which can thus be responsible for high rates of relapse after treatment, as well as for metastasis. Cancer stem-cell-targeting therapy thus represents a promising potential therapy for the treatment of breast cancer. In this study, we evaluated the role of CD44 in maintaining stemness and inhibiting the differentiation of BCSCs. Previous studies suggested that downregulation of CD44 allowed BCSCs to differentiate into cancer non-BCSCs or normal cells in breast tissue. To confirm this, we initially isolated BCSCs from malignant breast tumors based on their CD44 and CD24 expression pattern.

To determine the contribution of CD44 to the characteristics of BCSCs, we performed CD44 knockdown using a shRNA lentiviral vector and puromycin selection. This method was more effective than siRNA for generating a stable and pure cell population lacking CD44 expression, which could then be compared with non-knockdown cells.

The stemness of the CD44 knockdown cells was evaluated based on three criteria: the expression of genes related to stem cells, metastasis, and drug resistance; changes in the cell cycle; and the ability to form tumors *in vivo *in a NOD/SCID mouse model.

CD44 knockdown cells showed dramatically changed gene expression patterns compared with the original cells. Genes associated with the metastatic ability of cancer stem cells, especially *Muc-1*, *MMP9 *and *Myc*, were strongly reduced by CD44 knockdown. Mucin 1 is encoded by the *Muc-1 *gene. Mucin-1 protects the body from infection by binding pathogens to oligosaccharides via the extracellular domain, thus preventing the pathogen from reaching the cell surface [25,26]. Mucin-1 also functions in a cell-signaling capacity [27]. Over-expression of *Muc-1 *is often associated with colon, breast, ovarian, lung and pancreatic cancers [28]. Mucin-1 plays important roles in cancer development and metastasis by inhibiting the anti-tumor immune response [29], promoting the growth of cancer cells by binding to EGFR in an epidermal growth factor-dependent manner [30], preventing cell death by inhibition of p53-mediated apoptosis and promotion of p53-mediated cell cycle arrest [31], and promoting cancer metastasis [32-34]. Because Mucin-1 promotes the expression of *Myc *[35], levels of *Myc *expression were also decreased in association with Mucin-1 down-regulation, with consequent effects on the metastatic ability of BCSCs. A recent study by Fessler et al. showed that Mucin-1 was a determinant of trastuzumab (Herceptin) resistance in breast cancer cells, as well as being associated with resistance to taxol, doxorubicin, and cyclophosphamide [36]. Low expression of Mucin-1 would thus be expected to decrease metastasis and drug resistance in BCSCs.

EGFR and cyclin D1 expression were also reduced in CD44 knockdown cells. EGFR is often strongly expressed in many cancers [37,38], including breast cancer. However, BCSCs that weakly express this gene are unaffected by drugs that attack the EGFR, such as gefitinib, erlotinib, and cetuximab. The reduction of CD44 expression increased EGFR expression to a level similar to that in non-BCSCs, which are sensitive to chemotherapy. Cyclin D1 is encoded by the G1/S-specific CCND1 gene, and increased expression of cyclin D1 thus caused cells to move rapidly into S phase. However, cyclin E2 expression was not increased by CD44 knockdown, and cells were therefore mainly stopped in phase G1/S. The results of cell cycle analysis were in accord with these explanations.

Gene expression analysis also showed down-regulation of *Bcl-2 *by CD44 knockdown. Bcl-2 is capable of inhibiting anticancer drug-induced apoptosis mediated by the voltage-dependent anion channel in the outer mitochondrial membrane, and over-expression of *Bcl-2 *and *Bcl-XL *might confer resistance to chemotherapy [39]. Cells with low *Bcl-2 *gene expression are more sensitive to chemotherapy. Previous studies showed that CD44 knockdown cells were more sensitive to doxorubicin than BCSCs, similar to breast cancer cells [24]. FASN was also down-regulated in CD44 knockdown BCSCs. FASN expression is up-regulated in the early steps of breast cancer and represents a therapeutic target for breast cancer metastasis [40,41] and liposarcoma [42]. Inhibition of FASN suppressed the growth of cancer stem-like cells in breast cancer [43] and colon cancer [44], and induced apoptosis in diffuse large B-cell lymphoma [45] and in gastric-tumor-bearing mice [46]. CD44 knockdown was also associated with down-regulation of heat shock transcription factor 1 (HSF1) to a level similar to that seen in non-BCSCs. HSF1 is a major transactivator of genes coding for heat shock proteins. HSF1 is involved in tumor initiation, maintenance, and progression by regulating the expression of heat shock proteins [47]. Down-regulation of HSF1 decreased cell proliferation and enhanced sensitivity to hyperthermia in human melanoma cell lines [48]. It has thus been considered as a promising target for anti-cancer treatment [49], especially in breast cancer [50].

LEF1 up-regulates Oct4 promoter activity and physically interacts with Nanog; these comprise two key components of embryonic stem cell pluripotency. Down-regulation of LEF1 by siRNA induced differentiation of mouse embryonic stem cells [33]. The low expression of *LEF1 *in non-BCSCs and the down-regulation of *LEF1 *by CD44 knockdown indicated the differentiation of BCSCs into non-BCSCs. Moreover, *LEF1*, *TCF7 *and *Myc *are all members of the Wnt signaling pathway, and their down-regulation thus represents suppression of the Wnt signaling pathway. Similarly *Bcl-2*, *MMP7*, and *Myc *are members of the PI3K/AKT pathway; *HSF1*, *TP53*, and *Myc *belong to the Stress signaling pathway; and *PTCH1*, *PKRCE*, *PTGS2 (Cox-2)*, and *IL4R *are members of the Hedgehog, calcium and protein kinase C, and Jak-Stat pathways, respectively. Their reduced expression following CD44 knockdown demonstrated its effect on several signaling pathways. The Wnt, Hedgehog and Jak-Stat pathways are important pathways in stem cells and cancer stem cells and have been considered as promising therapeutic targets [51-54]. Down-regulation of some key signaling pathway genes proved that CD44 knockdown BCSCs underwent phenotypic changes from cancer stem cells to cancer cells or normal cells. Down-regulation of the Stress and calcium protein kinase C pathways might increase the sensitivity of CD44 knockdown BCSCs to some anti-tumor drugs, such as doxorubicin, because the Stress and protein kinase C pathways help cancer cells to cope with stress and changes of environment.

Changes in the gene expression profiles of CD44 knockdown BCSCs drove the cell cycle towards that seen in non-BCSCs. S-phase cells were increased and G2/M cells decreased in CD44 knockdown BCSCs and non-BCSCs compared with BCSCs. These cell cycle results resembled those found in cancer stem cells from solid tumors isolated on the basis of CD133 expression, showing that cancer stem cells were mainly in G2/M phase [55]. Karimi-Busheri et al. compared the cell cycles of mammosphere-forming and adhesive cells in cancer stem cells isolated from MCF-7 cell lines; most adhesive cells were in S phase, while mammosphere-forming cells were concentrated in the G2/M phase [56]. Thus, CD44 knockdown appeared to drive BCSCs toward a non-BCSC phenotype and differentiation.

The main physiological difference between cancer stem cells and the remaining cells in the tumor is their different tumorigenic potentials when transplanted into mice. Tumorigenic potential thus represents the gold standard for demonstrating a change in stemness of BCSCs. The tumorigenic potential of CD44 knockdown BCSCs in this study was reduced to that of non-BCSCs. Many previous studies found that as few as 50-100 BCSCs were adequate to generate tumors in NOD/SCID mice [3], while others found that 10^3 ^cancer stem cells could cause tumors [4,57]. In the current study, grafts of 10^3 ^BCSCs were capable of producing tumors in SCID mice, whereas CD44 knockdown BCSCs required at least 10^6 ^cells, similar to the situation for non-BCSCs. The altered biological characteristics of these cells indicated that CD44 knockdown changed the stem cell phenotype with high tumor-causing potential into cells with lower tumor-causing potential, representing differentiation of the cancer stem cells.

Differentiation therapy targeting cancer stem cells is currently under investigation by many groups, particularly focusing on the use of chemicals to cause stem cell differentiation. Takehara et al. showed that BCSCs differentiated when treated with acetaminophen, which also inhibited tumor formation in a nude mouse model [58]. Estrogen also causes BCSCs to differentiate, as demonstrated by a reduction in the number of cancer stem cells in tumors positive for estrogen receptors [59]. All-trans retinoic acid (ATRA) can prevent breast cancer recurrence by inducing BCSC differentiation and cell cycle arrest [2,60]. BCSCs may also be affected in terms of stem cell self-renewal, differentiation, motility and mesenchymal phenotype after treatment with the polyamine analog [1 N, 12 N] bis (ethyl)-cis -6.7-dehydrospermine (PG11047) [61]. Similarly, Roy et al. found that ATRA, trichostatin A and vorinostat caused dose-dependent decreases in the BCSC population, and showed that these differentiating agents reduced the number of BCSCs within the MCF7 cell line. Mammosphere formation in primary breast cancers (n = 3) was decreased by ≥ 25% by ATRA treatment combined with 6 Gy irradiation, compared with irradiation alone [62].

The results of the current study showed that CD44 plays an important role in the maintenance of BCSC stemness. Because inhibition of CD44 expression caused differentiation of BCSCs as well as reduced anti-tumor drug resistance [24], it is possible that gene therapy designed to interfere with CD44, as well as other factors that could reduce CD44 expression, represent promising therapeutic strategies for treating breast cancer, especially in combination with radiation or other anti-tumor agents.

This study used CD44 shRNA lentiviral particles to generate a stable CD44 knockdown BCSC population, with clear modifications in gene expression and cell characteristics. However, reverse-transcribed DNA can randomly insert into the cell genome and potentially disturb the function of cellular genes, leading to the activation of oncogenes and thus promoting the development of cancer. However, previous studies found that lentiviral vectors had a low tendency to integrate in places that caused cancer [63], and one study found no increase in tumor incidence and no earlier onset of tumors in a mouse strain following the use of lentiviral vectors [64]. In addition, we randomly evaluated 10 colonies and found similar results in terms of gene expression. Given that lentivirus integration is random, the 10 colonies would be exceptionally unlikely to contain similar insertion sites to disrupt the function of the genes.

## Conclusions

Down-regulation of CD44 caused changes in the phenotype of BCSCs. CD44 knockdown BCSCs lost the BCSC phenotype and showed reduced expression of genes related to stemness, metastasis and tumorigenesis, especially *Muc-1 *and *Bcl-2*. In addition, the cell cycle changed to resemble that seen in differentiated cells (non-BCSCs), and anti-tumor drug resistance and tumorigenic potential in NOD/SCID mice were both reduced. These data indicate that BCSCs were differentiated into non-BCSCs by CD44 knockdown. These results suggest that a combination of differentiation therapy aimed at down-regulation of CD44 in BCSCs, together with chemical or radiation therapies, represents a promising therapeutic strategy for the treatment of breast cancer, and also suggest that RNAi gene therapy could provide a novel differentiation strategy.

## Competing interests

The authors declare that they have no competing interests.

## Authors' contributions

All authors read and approved the final manuscript. PVP carried out studies including primary culture, isolation of breast cancer stem cells, gene expression analysis and designed the study and drafted the manuscript in cooperation with all other authors. NLCP and NTN performed the knockdown of CD44 by shRNA. NHT and TTD participated in the *in vivo *animal experiments. DVL and KDT participated in the cell cycle and flow cytometry analysis. NKP participated in designing the study and drafted the manuscript in cooperation with all other authors, and also checked PCR primers.
